# Healthcare workers’ beliefs, motivations and behaviours affecting adequate provision of sexual and reproductive healthcare services to adolescents in Cape Town, South Africa: a qualitative study

**DOI:** 10.1186/s12913-018-2917-0

**Published:** 2018-02-13

**Authors:** Kim Jonas, Rik Crutzen, Anja Krumeich, Nicolette Roman, Bart van den Borne, Priscilla Reddy

**Affiliations:** 1 School of Public Health and Primary Care (CAPHRI), Department of Health Promotion, Faculty of Heath, Medicine and Life Sciences, Maastricht University, P.O. Box 616, 6200 MD Maastricht, the Netherlands; 2 Division of Child and Adolescent Psychiatry, Adolescent Health Research Unit, University of Cape Town, Rondebosch, Cape Town, South Africa; 3 Department of Health, Ethics and Society, Faculty of Heath, Medicine and Life Sciences, Maastricht University, P.O. Box 616, 6200 MD Maastricht, the Netherlands; 4 Child and Family Studies, Faculty of Community and Health Science, University of the Western Cape, Cape Town, South Africa; 5 Population Health, Health Systems and Innovation Unit (PHHSI), Human Sciences Research Council (HSRC), Cape Town, South Africa

**Keywords:** Adolescents, Sexual and Reproductive Healthcare, Beliefs, Motivations, Cape Town, South Africa

## Abstract

**Background:**

Adolescents’ sexual and reproductive healthcare (SRH) needs have been prioritized globally, and they have the rights to access and utilize SRH services for their needs. However, adolescents under-utilize SRH services, especially in sub-Saharan Africa. Many factors play a role in the under-utilization of SRH services by adolescents, such as the attitude and behaviour of healthcare workers. The aim of this study therefore, was to explore and gain an in-depth understanding of healthcare workers’ beliefs, motivations and behaviours affecting adequate provision of these services to adolescents in South Africa.

**Methods:**

Twenty-four healthcare workers in public SRH services in Cape Town, South Africa participated in this qualitative study through focus group discussions. To fulfill the aims of this study, nine focus group discussions were conducted among the SRH nurses.

**Results:**

SRH nurses indicated that they are experiencing challenges with the concept and practice of termination of pregnancy. They explained that this practice contradicted their opposing beliefs and values. Some nurses felt that they had insufficient SRH skills, which hinder their provision of adequate SRH services to adolescents, while others described constraints within the health system such as not enough time to provide the necessary care. They also explained having limited access to schools where they can provide SRH education and pregnancy prevention services in the surrounding area.

**Conclusions:**

Nurses are faced with numerous challenges when providing SRH services to adolescents. Providing the nurses with training programmes that emphasize value clarification may help them to separate their personal beliefs and norms from the workplace practice. This may help them to focus on the needs of the adolescent in a way that is beneficial to them. At the health systems level, issues such as clinic operating hours need to be structured such that the time pressure and constraints upon the nurse is relieved.

**Electronic supplementary material:**

The online version of this article (10.1186/s12913-018-2917-0) contains supplementary material, which is available to authorized users.

## Background

Whilst adolescents’ sexual and reproductive healthcare (SRH) needs have been prioritized globally, many of these needs remain unmet [1, 2]. SRH services for adolescents include, services such as family planning (FP) counseling, and services; prenatal and postnatal care and delivery; termination of pregnancy (TOP); post abortion care (PAC); treatment and prevention of sexually transmitted infections (STIs) including HIV; and information and counseling services regarding human sexuality [3]. Adolescents have the rights to fully access and utilize these services as emphasized and supported by the United Nations Population Fund Agency and the World Health Organization (WHO) [3, 4]. The availability and use of these services are essential to improve the SRH outcomes, as adolescents typically tend to engage in behaviours that place them at risk, for example risky sexual experiences [5, 6]. However, adolescents under-utilize SRH services, particularly in sub-Saharan Africa (SSA) [1–6]. The high rates of teenage pregnancy and unsafe abortions in South Africa (SA) have been attributed to the poor use of SRH services, among other determinants [7–10]. Despite the supportive policies and guidelines in SA, the utilization of SRH services by adolescents is described as less than optimal [7, 11–13].

Factors that contribute to the poor use of SRH services include the unfavourable behaviours and attitudes of healthcare workers towards adolescents seeking the services [11–16]. Healthcare workers’ behaviour discourages young people from attending clinics and keeping follow-up appointments [17, 18]. Lack of respect for adolescents’ privacy and previous ill treatment by healthcare workers were some of the reasons that discouraged sexually active adolescents to seek SRH services [17]. Studies in SA have demonstrated that a lack of skills necessary for providing services to adolescents, as well as untoward social norms towards adolescent sexual practices, result in poor treatment of the pregnant teenage girl [8, 19]. The resultant unfavourable behaviours and attitudes towards pregnant teens contributes to serious barriers in them seeking health care from the available SRH services.

Self-efficacy to provide family planning (FP) and maternal and child healthcare (MCH) services to adolescents were strongly associated with intentions to provide the services [8]. Furthermore, the limited SRH skills and lack of confidence of some nurses in providing the services to adolescents exacerbates the problems faced by adolescents [7, 8, 13, 19]. Studies into the reasons for these unfavourable behaviours and attitudes show that the way healthcare workers conduct themselves when providing healthcare services is usually informed by their beliefs and or values [15].

A youth friendly service environment, which could improve access to and utilization of SRH services, would require nurses and other healthcare workers to demonstrate attitudes and engage in behaviours that are supportive, caring and inviting. This in turn may help reduce the high rates of teenage pregnancies and unsafe abortions among adolescents in SA.

An in-depth understanding into the determinants of healthcare workers’ unfavourable behaviours and attitudes is needed to develop interventions that may contribute to an enabling environment where adolescents can fully access and utilize the SRH services. The findings from this research will contribute to developing programmes and policies that would improve healthcare workers’ behaviors and attitudes, as well as adolescent health care seeking behavior.

The aim of this study was therefore to explore and gain an in-depth understanding of professional healthcare workers’ beliefs, motivations and behaviours affecting adequate provision of SRH services to adolescents in SA. The data gained will help identify where to intervene to eliminate healthcare workers’ unfavourable behaviours and attitudes. In addition, this data will provide ideas on how to change unfavourable behaviours and attitudes of healthcare workers and encourage adolescents to access and utilize SRH services.

## Methods

A qualitative study was conducted among healthcare workers at public clinics situated in the urban centres of Cape Town. The facilities included the Primary Health Clinics (PHCs), and the Community Health Centers (CHCs). PHCs are defined as facilities that provide a range of primary health care services, which are usually open for 8 h or more a day based on the need of the community to be served. CHCs are defined as facilities that usually provide more extensive services than provided by the PHCs, with 24-h maternity, accident and emergency services and beds where health care users can be observed for a maximum of 48 h and which normally have a procedure room but not an operating theatre [20]. Both PHCs and CHC typically provide similar services to adolescents, except in the case where the services are not being provided in PHCs. For example, termination of pregnancy services are not offered in some PHCs, so these PHCs refer to CHC and other secondary levels of care for such services.

### Theoretical framework

The Reasoned Action Approach (RAA) was used as a guide and framework for the development of the questions about the determinants of behaviour, following a belief elicitation procedure [21]. RAA essentially entails beliefs associated with performing or not performing a particular behaviour. For example, when an individual holds beliefs about anticipated negative consequences of performing the behaviour, it is likely that the individual will not perform the behaviour. These beliefs determine ones’ attitude towards performing or not performing the behaviour [21].

### Sampling

The sampling framework for healthcare facilities consisted of public healthcare facilities that provided SRH services in the district. There were 46 clinics in Cape Town, of which 13 were the CHCs and 33 were PHCs. Not all these facilities provided SRH services, but all CHCs did. The selection of clinics was therefore based on the clinic having to provide SRH services, the community profile that the clinic was serving in order to have views from the different socio-economic status (SES) of the Cape Town communities, and, lastly, the feasibility to conduct the study (e.g., in terms of logistics).

Thus, facilities were selected purposively in order to meet the objectives of the study. Twelve clinics were selected for the study, but two clinics were deemed unsafe to be visited due to violent protesting not related to this study in the community, and were therefore excluded. The final sample for the study consisted of ten clinics. Eight were classified as PHCs and two as CHCs. All clinics were based in the urban settings of Cape Town. Four clinics were located in the higher SES neighbourhoods while the other six clinics were in the lower SES. Nine focus group discussions (FGDs) were conducted for this study.

### Participants

A total of 28 professional healthcare workers who worked in SRH services were purposively approached face-to-face and recruited from the clinics visited (with help from the facility managers). They were then invited to participate in the FGDs that were conducted within the facility that they worked at. Of these, 24 of them agreed and participated in the study. Four participants refused to participate because of work constraints such as time and patient overload. The participants were diverse in terms of demographic characteristics (see Table 2).

It must be noted also that healthcare workers in SRH services had no specific training or incentives to provide services to adolescents. Their education consisted of the usual general nursing curriculum with no speciality courses in adolescent health and behaviour. Although training and refresher courses on SRH services including family planning are offered throughout the year, none of these were specific to adolescent health.

The City of Cape Town municipality approved the conduct of this study on condition that the clinic operations would not be disturbed. Furthermore, the researchers had to ensure that patient care would not be disrupted in any way by the study’s activities. In order to ensure that the clinic operations were not disrupted, it was agreed that only a portion of the healthcare workers in a specific clinic could participate in the study. This meant that in some clinics; depending on patient load relative to the number of healthcare workers in a facility, only 2 participants could be made available for the study. The possibility of conducting the FDGs after hours was explored but this was not an option for the healthcare workers as they had family responsibilities to fulfill. Two participants in such cases were engaged in an in-depth discussion albeit not a focus group discussion. This provided time for a deep exploration into the topic of concern for this study. Therefore, due to these circumstances, including the general shortage of professional healthcare workers in public healthcare facilities in SA, and especially SRH designated healthcare workers; two to four participants in a FGD were acceptable, where in-depth discussions were used with two participants.

### Data collection tools

Open-ended questions with probes were used to guide the focus group discussions and the in-depth discussions. A brief questionnaire to document nurses’ socio-demographic characteristics was also used. The belief elicitation procedure of Fishbein and Azjen [21] was used to develop the guide, which enabled the exploration of beliefs, motivations and behaviours of healthcare workers serving adolescents in providing standard care as per the South African SRH guidelines. The guide was considered an FGD guide as it was intended for use in a group of two or more participants. Appendix 1 provides an overview of statements and questions included in the FGD guide (see Additional file 1).

### Procedure

To ensure that the FGD guide was applicable and the questions were understandable to participants, two pilot FGDs were conducted with healthcare professionals in SRH services. The guide was carefully modified to include the language and content gained during the pilot. This was done to improve the face validity of the guide and probing questions. For example, after the pretest, this question [Are there certain situations/circumstances (motivations) where you would NOT provide sexual and reproductive healthcare services to adolescents, like family planning? Please explain] was modified in this manner: “Healthcare workers approve of adolescents using contraceptives if they are sexually active. Probe for further details.”

Two authors (KJ and RC) co-facilitated the FGDs. Both KJ (female) and RC (male) are experienced researchers with RC having obtained a PhD and KJ a Masters degree at the time of data collection. A research assistant who was trained for this specific study was always present during the FGDs and took notes. There was no existing relationship with participants prior to data collection for this study and therefore no conflict of interest between the participants and the researchers. All FGDs were conducted at the clinics and were audio-recorded with hand-written notes taken during the discussions. Data saturation was discussed after the sixth FGD and it became clear that there were no new views transpiring from the FGDs by the ninth FGDs and data was deemed saturated at this point.

### Data analysis

Data was analyzed using thematic analysis. The data analysis followed the Tesch’s eight steps for coding and analysing qualitative data, which are: 1) careful reading of all transcripts, 2) thorough examination of all FGD transcripts and making notes on the side, 3) produce a list of transpiring topics, 4) code the data using the abbreviations of the topics, 5) categorize the topics using the most descriptive wording, 6) alphabetically abbreviate each category, 7) assemble the data according to the categories, and 8) recode data if needed. The focus group discussions data was transcribed verbatim from the audiotape recordings, and analysed. The ages of participants were grouped into 5 categories with a 5-year gap in between (1 = 25–30, 2 = 31–36, 3 = 37–42, 4 = 43–48, 5 = 49 and above).

Typed transcripts were read several times, and codes were developed and defined based on the objectives of the study, thus themes were derived from the data. Two independent researchers (KJ and RC) developed and checked the coding until they reached consensus. Then, codes were grouped into sub-themes and then into themes. Data was coded and analyzed using *Nvivo* qualitative data analysis software. The coded transcripts were analyzed by running query reports and primary document tables of themes and sub-themes, to explore the issues from the various FGDs as shown in Table 1. Unfortunately, transcripts were not returned to participants for comment and or correction due to a number of limitations including resources.

**Table 1:** Generating Themes

Probe questions	Quotes	Sub-themes	Themes
Do you think your own personal opinions (feelings) about adolescents’ sexual activity have an effect on how you deal with adolescents seeking sexual and reproductive healthcare services, like family planning? Please elaborate	“Its better they use family planning than getting unwanted pregnancy in my opinion.” **Certificate qualified nurse**	-Reduce teenage pregnancy	Nurses’ motivations
	“We cannot run away from that reality, we advise, we say be careful we give condoms, come for HIV testing but we can’t run away from that people are gonna have sex,…” **Diploma qualified nurse**	-Reduce STI and HIV rates -Nurses personal views	Nurses’ beliefs and personal views
	“But I’ll admit I do tend to discourage pills because I think they are more likely to miss their tablets. Not all of them do, but they’re more like to forget to take the pill.” **Degree qualified nurse**	-Nurses personal views and professional duties	
	“It doesn’t matter if I don’t agree with termination of pregnancy, if that person is coming for those services; it is my duty as a professional nurse to provide what I’m supposed to do.” **Degree qualified male nurse**	-Nurses beliefs and personal views on TOP	
	“it’s very sad you know it touches my heart, so that is the only thing [termination of pregnancy] in nursing that is actually impractical for me you know.” **Diploma qualified nurse**		
Are there any obstacles or reasons for you, personally that discourage (prevent) you from providing sexual and reproductive healthcare services to adolescents? E.g. Religious or cultural beliefs?	“Now my belief says, if I give condoms, I’m actually encouraging that child to go have sex. If the child isn’t sexually active, then I’m saying okay, take the condom and you can start.” **Diploma qualified nurse**	-Nurses views on policy guidelines in ASRH services	Nurses’ beliefs and personal views
	**“**I do not feel comfortable dealing with teen coming for termination of pregnancy, I just refer to another sister.” **Diploma qualified nurse**	-Nurses personal views on TOP	
	**“**I’m not pro-abortion, I feel that its murder but I will discuss that at home with my family when I’m here someone walk through that door all I see is someone who is desperate for help…” **Diploma qualified nurse**	-Nurses personal views on TOP	
	“From my side it would be very difficult for me to give somebody a referral letter [for termination of pregnancy], just because I am a born again Christian.” **Diploma qualified nurse**	-Nurses personal views on TOP	
Do you think certain things/circumstances in the facility (obstacles) facilitate the situations where you will NOT provide SRH services?	“Sometimes lack of equipment, it actually hinders our work… for example, now we don’t have the reproductive health form, we out of stock” **Degree qualified nurse**	-Limited SRH resources	Challenges in SRH services
	“Shortage of staff.” **Diploma qualified nurse**	-Staff shortage	
	“…but if anything happens in the sense that there’s no staff or something happens, they will need me or her to help the other patients in other sections then my patients will be sent home or my patients will be cancelled for the day because that is not a priority.” **Diploma qualified nurse**	-Staff shortage	
	“And its worse if you get a newly diagnosed HIV patient, you have to spend an hour with the patient, oh that one is worse, it actually gets to you… Sometimes you even leave a patient feeling like maybe I didn’t do enough, but because you have to see others waiting for you and there’s no time.” **Degree qualified nurse**	-Time pressure	
	“Its just like you say next, and then next, so you don’t really give attention and information. For example if they want to change the method, you don’t ask why you just say ok, and just give, while if you have time you would have dealt with her better. Then you feel like 10 is overwhelming from 3-4 pm and you just rush, you asking the color of discharge, when did you start experiencing the symptoms, you not even looking at her you writing because you think what’s important is to treat, not think you must reiterate because you rushing. You do like rush-rush with them, because you don’t have time, due to the short staff so it does happen.” **Degree qualified nurse**	-Quantity over quality	
	“I have done the course [family planning course] but also I do not have the implanon training so I can’t insert it so sometimes they come and want the implanon and maybe she’s [the nurse who inserts implanon] not in so now you have to tell them there’s no one to do it at that moment, so we have to give them another appointment.” **Diploma qualified nurse**	- SRH skills	
	**“**And the patients only come after school and most schools closes at 3 pm so they come here after 3, and there’s only an hour to see more than 30 patients and we are only 3 nurses, so there is no time to do the talk.” **Diploma qualified nurse**	-Time pressure	
	**“…**We are struggling to get family planning services in some high schools around the clinic, and most of the teenagers who are coming for TOP are coming from these schools we are not allowed to go to… And that shows that these schools need our FP outreach services…” **Diploma qualified nurse**	-School access	
Are there any reasons for you, personally that encourage (promote) you to provide sexual and reproductive healthcare services to adolescents? For example, what makes you more willing (motivates you) to provide sexual and reproductive healthcare services to adolescents?	“There is a lot of teenage pregnancies because I see a lot of pregnant girls and that worries me because these girls’ future is ruined… she might not be able to go back to school and finish, depending on the family situations… some girls’ schooling really end with their pregnancy and now she has to go find a job, any job because she is not educated…but if she was on family planning that would not happen. That is why I am happy when I see the girls on family planning, I want to help them not get pregnant.” **Diploma qualified nurse**	-Nurses concerns for adolescents’ future -Reduce teenage pregnancy	Nurses motivations
	“Sometimes there are lots of babies who are dumped, the unexpected pregnancy, the problems we see in our communities, we see those babies who are not having parents, the struggling families, the babies who don’t have parents. This teenager that is not at school now because she got pregnant and had to dropout.” **Certificate qualified nurse**	-Reduce unwanted pregnancy and TOPs -Nurses concerns for adolescents’ future	
	“We actually want to decrease the number unplanned pregnancy and abortions…” **Diploma qualified nurse**	-Reduce unwanted pregnancy and TOPs	
	“What motivates me is the rate of HIV and the rate of teenage pregnancy, and that of STIs, so that makes me want to help them, to educate them, make them understand…” **Diploma qualified nurse**	-Reduce STIs and HIV rates	
	“Personal experiences, you’ve got someone at home who’s infected with HIV, your own family member got pregnant as a teenager, so you look into yourself and your experiences.” **Degree qualified nurse**	-Personal experiences	

To strengthen the trustworthiness of the data, data triangulation was conducted. Written notes and observations during data collection were performed by the research team and these were compared with the data from FGDs and corroborated the findings of this study. Furthermore, more than one person (KJ and RC) performed data analysis independently and analyses were compared. The two researchers met regularly to compare and discuss their findings until consensus was reached. All these efforts were taken to increase credibility and trustworthiness of the study findings.

## Results

### Participants

Table 2 shows the demographic characteristics of the nurses who participated in the FGDs. The ages of these nurses ranged from 25 to 56 years old. Most of the women were either married or living together with their partner. Men also participated in the study, although only very few, and all of them were married. The majority of participants had a diploma in nursing and only a few had a degree in nursing. A diploma qualification is obtained through colleges and often takes up to 3 years, while degree is a university obtained qualification, which takes 4 years and includes training in extensive clinical skills. The certificate in nursing is lower than the diploma, with not much practical experience and skills of nursing care. Very few nurses in this study only had the certificate qualification of nursing. This is also reflected in their current position where a small number of them were just nursing sisters, which actually means that they are nursing assistants, as they do not have advanced nursing skills and training. A large number of them were enrolled nurses followed by registered nurses, and very few were specialized registered midwives (see Table 2 below).

**Table 2:** Demographic characteristics of the nurses

Demographics Characteristics	
	N (%)
Gender	
Male	3 (12)
Female	21 (88%)
Age group	
1 (25–30)	4 (17%)
2 (31–36)	5 (21%)
3 (37–42)	4 (17%)
4 (43–48)	3 (12%)
5 (49+)	8 (33%)
Marital status	
Married	13 (54%)
Single	5 (21%)
Divorced	3 (13%)
In a relationship / Cohabitating	2 (8%)
Widow	1 (4%)
Race	
Black African	14 (58%)
Coloured	8 (34%)
Indian	1 (4%)
White	1 (4%)
Education	
Certificate in nursing	2 (8%)
Diploma in nursing	13 (54%)
Degree in nursing	9 (38%)
Current position	
Nursing sister	5 (21%)
Enrolled nurse	11 (46%)
Registered nurse	6 (25%)
Registered midwife	2 (8%)
Total	24 (100%)

**Fig. 1:**
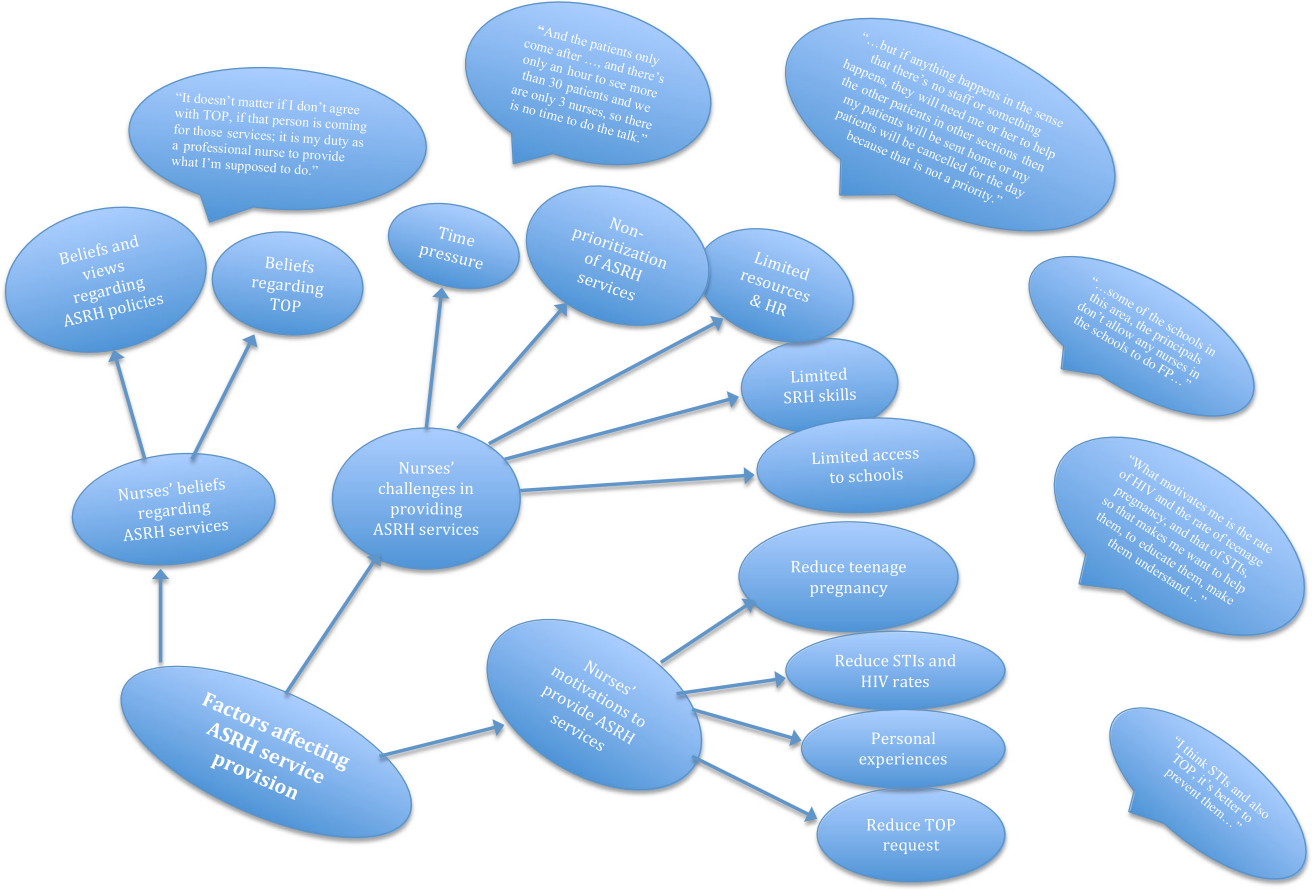
Overall themes. Key: ASRH = Adolescents Sexual and Reproductive Healthcare, TOP = Termination of Pregnancy, FP = Family Planning

The results of the FGDs are presented below according to the following themes: nurses’ beliefs and views of adolescents’ SRH services, nurses’ motivations to provide SRH services to adolescents, challenges hindering the nurses’ provision of adequate SRH services to adolescents, and finally, nurses’ suggestions for the improvement of access to and utilization of SRH services by adolescents. Figure 1 shows an overall picture of the themes produced during data analysis.

### Nurses’ beliefs and views of adolescents’ SRH services

When discussing how the nurses viewed the adolescents’ SRH services consequently brought up a conflict between nurses’ underlying beliefs regarding the use of FP, including TOP by adolescents and their professional code of conduct. With regards to TOP, quite a few nurses had strong feelings against this basic, yet necessary component of SRH services for adolescents. The strong feelings were associated with the nurses’ beliefs, which were against TOP for many nurses. However, on the other hand nurses also felt that it is their duty to provide the services irrespective of their own beliefs; otherwise they will be in conflict with their professional code of conduct. Although this was clear for many nurses, some still felt that they cannot provide TOP services to adolescents or even to adults because of their own beliefs, regardless of their professional responsibility to render the services required of them.

*“It doesn’t matter if I don’t agree with termination of pregnancy, if that person is coming for those services; it is my duty as a professional nurse to provide what I’m supposed to do.”* ***Male nurse, degree qualified***

***“*** *I’m not pro-abortion, I feel that its murder but I will discuss that at home with my family when I’m here someone walk through that door all I see is someone who is desperate for help and I have to help her as much as I can…”* ***Diploma qualified nurse***

*“No, I will never do that part [termination of pregnancy], never! I am not ready to deal with that part… my religious beliefs are against it, but also my personal beliefs.”* ***Diploma qualified nurse***

Nurses felt that the SRH services for adolescents they are expected to provide are sometimes in conflict with their own beliefs and values. According to the nurses, it appeared that certain components or guidelines of the SRH services, which they have to follow when providing the services to adolescents, are contradictory to their own traditional and personal beliefs. Nurses used the provision of condoms as an example, whereby they felt providing a condom to a 12 year old is not appropriate because they think this is encouraging the teenager to start having sexual intercourse.

*“Now my belief says, if I give condoms, I’m actually encouraging that child to go have sex. If the child isn’t sexually active, then I’m saying okay, take the condom and you can start.”* ***Diploma qualified nurse***

Furthermore, the nurses reported a shifted focus in the policies and guidelines they follow when serving their adolescent clients. For example, the nurses stated that abstinence; a previously recommended and emphasized element in the SRH guidelines has now been sidelined. The nurses expressed the need to have the abstinence element re-instated in the prevention strategy for STI and HIV management.

“*Like we don’t speak about abstinence anymore, you don’t even see it on posters, its not even spoken of now and its just condoms and condoms.”* ***Degree qualified nurse***

### Nurses’ motivations to provide SRH services to adolescents

In the quest to understand what motivates the nurses to provide SRH services to adolescents, the nurses were asked to share their motivations. Interesting and positive responses came up, mostly in support of better quality SRH services for adolescents. The nurses’ concerns regarding the high teenage pregnancy rates among adolescents in Cape Town were common motivations for all the nurses. The nurses also had concerns regarding the adolescents’ future, which motivated them to provide the services to them so that they can finish school for example, instead of falling pregnant and ruin their future. From the FGDs, it was clear that nurses want to help decrease the teenage pregnancy rates.

“*There is a lot of teenage pregnancies because I see a lot of pregnant girls and that worries me because this girl’s future is ruined… she might not be able to go back to school and finish, depending on the family situations… some girl’s schooling really end with their pregnancy and now she has to go find a job, any job because she is not educated…but if she was on family planning that would not happen. That is why I am happy when I see the girls on family planning, I want to help them not get pregnant.”* ***Diploma qualified nurse***

The prevention of TOP also came up from some of the FGDs. Nurses appeared to be strongly motivated to prevent unwanted pregnancies, as well as abortions among adolescents.

*“We actually want to decrease the number of unplanned pregnancy and abortions…”* ***Diploma qualified nurse***

*“Lots of unwanted babies and TOPs, that makes me want to help them… I wish all of them can be on FP you know”* ***Diploma qualified nurse***

Another important motivation for the nurses to provide SRH services to adolescents was to reduce the high rates of STIs and HIV infection among adolescents. Additionally, some nurses were inspired by their very own personal experiences as teenagers to serve SRH services to adolescents.

*“What motivates me is the rate of HIV and the rate of teenage pregnancy, and that of STIs, so that makes me want to help them, to educate them, make them understand…”* ***Diploma qualified nurse***

*“Personal experiences, you’ve got someone at home who’s infected with HIV, your own family member got pregnant as a teenager, so you look into yourself and your experiences.”* ***Degree qualified nurse***

### Challenges hindering the nurses’ provision of adequate SRH services to adolescents

In this study there was also a quest on exploring what then hindered the nurses to provide adequate SRH services to adolescents. The challenges the nurses face in these services has a potential to influence their behaviour in general, hence exploring challenges faced by the nurses was necessary. Among the many challenges nurses were faced with, time pressure was their most serious challenge in SRH services. Many of the nurses perceived the healthcare system as in favour of quantity over quality services, forgetting that different ailments need different time-allocations and this made the conditions they work under difficult to cope with.

*“Its just like you say next, and then next, so you don’t really give attention and information. For example if they want to change the method, you don’t ask why you just say ok, and just give, while if you have time you would have dealt with her better. Then you feel like 10 patients is overwhelming from 3-4pm and you just rush, you asking the color of discharge, when did you start experiencing the symptoms, you not even looking at her you writing because you think what’s important is to treat, not think you must reiterate because you rushing. You do like rush-rush with them, because you don’t have time, due to the short staff so it does happen.”* ***Degree qualified nurse***

*“And its worse if you get a newly diagnosed HIV patient, you have to spend an hour with the patient, oh that one is worse, it actually gets to you… Sometimes you even leave a patient feeling like maybe I didn’t do enough, but because you have to see others waiting for you and there’s no time.”* ***Degree qualified nurse***

In addition to the time pressure, nurses also expressed the lack of resources as another challenge hindering the adequate provision of SRH services, together with the shortage of staff in the clinics. Furthermore, the nurses felt that the healthcare system does not prioritize the SRH needs of adolescents. When the clinics are very busy or when there is an emergency in the clinic, the nurses would be deployed to the units with high demand, leaving the SRH services not functional for that day. Moreover, the nurses expressed their limitations with certain specific SRH skills and training, such as the insertion of intra-uterine devices (IUDs) and the contraceptive implants.

*“Sometimes lack of equipment, it actually hinders our work… for example, now we don’t have the reproductive health form, we out of stock ”* ***Degree qualified nurse***

*“…but if anything happens in the sense that there’s no staff or something happens, they will need me or her to help the other patients in other sections then my patients will be sent home or my patients will be cancelled for the day because that is not a priority.”* ***Diploma qualified nurse***

*“I have done the course [FP course] but also I do not have the implanon training so I can’t insert it so sometimes they come and want the implanon and maybe she’s [the nurse who’s trained to insert implanon] not in so now you have to tell them there’s no one to do it at that moment, so we have to give them another appointment.”* ***Diploma qualified nurse***

Nurses also reported some external factors impacting on the provision of adequate SRH services to adolescents. These factors included limited access to schools in the areas surrounding the clinic. The nurses from the clinics in the higher SES setting experienced more challenges in gaining access to the schools, while nurses from the clinics in the lower SES settings did not experience such challenges, except for one school in this setting. According to the nurses who worked in the higher SES settings, they find this challenge enormously frustrating for their FP outreach programme to adolescents.

***“…*** *We are struggling to get FP services in some high schools around the clinic, and most of the teenagers who are coming for TOP are coming from these schools we are not allowed to go to… And that shows that these schools need our FP outreach services…”* ***Diploma qualified nurse***

### Nurses’ suggestions for the improvement of access to and utilization of SRH services by adolescents

After sharing their motivations, frustrations and the challenges the nurses face when providing SRH to adolescents, they had a few suggestions that they believed would make their working environment better and improve access to and utilization of these services by adolescents. The nurses in this study believed that a separate room or space for adolescents’ SRH services, as well as a youth-friendly environment would improve access to and utilization of the services. The nurses also felt strongly that the operating clinic hours are not suitable for the adolescents and therefore need to be extended by at least 30 min to an hour to accommodate the adolescents that finish school at 3 pm and have to travel to the clinic. Some nurses recommended having some services removed from the SRH service package of adolescents, such as TOP services. Lastly, some nurses also wished to bring the services to adolescents, in their schools.

***“*** *I think if there can be a slot longer than 16:30, like maybe the clinic say we closing for youth at 17:30, just to accommodate those who are unable to come a bit earlier.”* ***Degree qualified nurse***

***“*** *If they could prevent or stop TOP at all. We really should not be having so many TOP sites and TOPs, because TOPs to me its too much they should not be so many… I would rather not have teenagers going for TOP; it should not be an option.”* ***Degree qualified nurse***

*“… if the school governing body can allow us to go to the schools. Because in the clinic they come pregnant but in the schools we can do the prevention at school.”* ***Degree qualified nurse***

## Discussion

This study aimed to explore beliefs, motivations and behaviours of professional healthcare workers affecting adequate provision of SRH services to adolescents. The nurses in this study seem highly motivated to serve adolescents with SRH services, although they are struggling with their personal norms and values regarding adolescents’ SRH needs and services. According to the FGDs however, a combination of factors interfere with their motivation and makes it challenging for them to provide adequate quality SRH services to their adolescent clients. These factors include: their own norms and values with regards to adolescents’ SRH services, the current guidelines and policies they have to follow when serving SRH services to their adolescent clients, and the healthcare systems’ related factors including time constraints. All these factors play a crucial role in adequate provision of SRH services and how the nurses behave under these challenges, and that potentially affects provision of adequate SRH services to adolescents.

One such challenge nurses reported to have difficulties with is TOP because of their own norms and values with respect to TOP. For example, some nurses reported their religious and personal beliefs to be against TOP, which makes it difficult for them to provide this service to the adolescent in need. The nurses even went on to state that they would try to persuade adolescents to keep the baby instead of terminating the pregnancy, compromising the adolescents’ rights to obtaining the services they need. Furthermore, some nurses suggested that TOP services should be totally removed from adolescents’ SRH services. Unfavourable behaviours and attitudes of healthcare workers towards TOP have been reported elsewhere as well [2, 22, 23]. This undermines the adolescents’ rights to adequate quality SRH services, including safe TOP as stipulated by the WHO and the United Nations Population Fund Agency [3, 4]. Denying adolescents TOP not only violates their rights to SRH services, but also contributes to the high teenage pregnancy and to unsafe abortions among adolescents. It is clear that nurses in SRH services still have challenges with TOP, despite its’ long existing legalization in the country. This finding is similar to other findings in sub-Saharan Africa where some nurses had negative attitudes towards TOP in general and in particular for adolescents [1, 22]. Healthcare workers are reported to often act as a barrier to care by failing to provide young people with supportive, nonjudgmental, youth-appropriate services [23]. Consequently, the barriers adolescents face to accessing and utilizing SRH services for their needs result in delayed care-seeking, under-utilization of services, unintended and unwanted pregnancies, and unsafe abortions [7, 11–13, 22, 23].

The nurses’ challenges with TOP are coupled with their self-reported limited skills in providing SRH services such as the insertion of modern methods of FP like the contraceptive implant. SRH skills are important in delivering adequate services to adolescents. For example, low self-efficacy among nurses in conducting some SRH services to adolescents in SA has been reported, and this affects provision of adequate quality services [8]. Thus, the limited skills among the nurses may also act as a barrier to adolescents’ access to and utilization of SRH services as they reported that some adolescents are turned away when the skilled nurse is not available to provide the service to them. A study in Johannesburg also reported similar findings, where several nurses reported not to be trained to insert the IUDs [24]. In order to adequately provide SRH services to adolescents, nurses in these services need to be fully trained and equipped to provide the services and handle all adolescent SRH issues. If the nurses possess the necessary skills in adolescents’ SRH services given their motivation, it is highly likely that they may provide the required services adequately, as skills are an important predictor of service provision [8]. Therefore, efforts to increase training among healthcare workers in SRH to improve their SRH skills are needed. The fact that the current training and refresher courses are not mandatory and incentivised might be one of the reasons behind the limited SRH skills among the nurses and thus, needs to be addressed. Incentives may encourage and improve participation of healthcare workers in these trainings, while concurrently improving their knowledge and skills of SRH services.

Furthermore, the nurses reported time pressure which does not only affect adequate provision of SRH services to adolescents, but also affects the quality, access to, and utilization of the services by adolescents, as well as nurses’ behaviour when under pressure. Because the nurses are under pressure with time, they end up rushing through the services and that often results in poor quality services. The quality of SRH services adolescents receive is an important factor in deciding whether or not to ever seek the services again, and it is unlikely that an adolescent who received poor quality service would be enthusiastic in seeking the services in future. Time pressure therefore affects the nurses’ provision of quality SRH services and potentially hinders access to and utilization of the services by adolescents [11, 12, 19]. In all the FGDs, nurses reported that the operating clinic hours are not convenient for some of the school-going adolescents. Moreover, the time allocated for them on those clinics that have a youth clinic day(s) is insufficient to adequately provide quality SRH information and services to adolescents. This healthcare systems related challenge is a contributing factor to the poor access to and under-utilization of SRH services by adolescents, as they may not be able to reach the clinic during the clinic operating hours after school. This finding echoes findings from another study in SA where healthcare workers suggested extended clinic hours to accommodate school-going youth who are not catered for in the “current” clinic operating hours [24].

The reported non-prioritization of SRH services in the clinics, which substantially hinders provision of the services to some adolescents is concerning. It is worth noting that prioritization of SRH services are not the nurses’ responsibilities, but that of the healthcare system itself. In addition to this non- prioritization, nurses added the shifted focus in the guidelines and policies as another healthcare system related challenge they experience. Both these healthcare systems’ related challenges affect the nurses’ behaviour with respect to providing adequate quality SRH services to adolescents and the adolescents’ access to and utilization of SRH services. According to the nurses, whenever there’s an emergency or shortage of staff in other sections of the clinic, the nurses have to turn away or cancel their adolescent clients coming for SRH services that day so that the nurses can go where there are “serious” emergency cases. This finding is a cause for concern and is in conflict with the global efforts and strategies to prioritize access to high quality SRH services for adolescents [3, 4, 23]. Furthermore, some nurses reported that both the shifted focus of the guidelines and the provision of condoms to every adolescent coming for SRH services is challenging for them. Indeed, both the National contraception, fertility planning policy guidelines and the National Contraception Clinical Guidelines place an emphasis on the dual protection which is the provision of condoms in addition to the contraceptive method provided [25, 26]. This leaves the nurses with limited room to discuss and encourage abstinence to their adolescent clients, while they appreciated the opportunity before as it enabled them to better manage their own norms and values. These findings show the complexity of the nurses’ situation in SRH services, which hinders them to provide adequate quality SRH services to adolescents, as well as the barriers to accessing and utilizing SRH services by adolescents.

This study only studied nurses providing SRH services in a big urban city and not in rural areas. In rural areas, working conditions for providing the same services are substantially different from those in the big cities. Therefore, the results of this study are limited to the urban areas, and that is to be taken into account when interpreting the findings. A similar study would need to be undertaken in rural areas to compare our findings. Despite this limitation, the findings of this study provide sufficient information regarding the factors affecting adequate provision of sexual and reproductive healthcare services to adolescents in Cape Town, as well as areas of improvement in order to increase access to and utilization of the services. This study adhered to the consolidated criteria for reporting qualitative research (COREQ): a 32-item checklist for interviews and focus groups [27].

To our knowledge, this study is the first study to investigate healthcare workers beliefs and behaviours in order to understand challenges affecting adequate provision of sexual and reproductive healthcare services in SA.

### Implications for practice

Interventions to avert unfavourable behaviours and attitudes of healthcare workers, especially towards TOP need to be intensified. Such interventions may include ‘value clarification training’ among the nurses in these services to eliminate negative attitudes towards TOP services at large [24]. Value clarification training might help address the nurses’ beliefs by clarifying their roles as healthcare providers and how bringing their beliefs into their work may affect adequate provision of the services, leaving the clients’ needs unmet. A few studies that applied this type of intervention reported positive results within the South African context, such as improved attitudes towards termination of pregnancy [24–26, 28–30]. On a policy level, prioritization of adolescents’ SRH services requires attention. Perhaps a specifically dedicated adolescent’ SRH nurse, who will always be available to attend to the adolescents’ SRH needs, irrespective of other emergencies in the facility, is indeed needed to meet the adolescents’ SRH needs in SA. This study acknowledges the challenges the healthcare system of SA is experiencing in terms of human resources for public healthcare services. However, there is a need to revisit the quantity over quality principle. The expectations that the nurses in these services assist at least 30 clients a day hinders the provision of adequate quality SRH services and information to support adolescents in making informed decisions regarding their SRH needs. Furthermore, enough time spent with adolescent during SRH consultations may help build a good rapport and consequently improve the adolescent-nurse relationship, and access to and utilization of SRH services.

## Conclusion

Professional healthcare workers are faced with numerous challenges when providing SRH services to adolescents. These include their conflicting personal norms and values with regards to adolescents’ SRH services, the current SRH guidelines and polices, and the healthcare systems related factors such as time constraints. Providing the nurses with training programmes that emphasize value clarification may help them to separate their personal beliefs and norms from the workplace practice. This may help them to focus on the needs of the adolescent in a way that is beneficial to them. At the health systems level, issues such as clinic operating hours need to be structured such that the time pressure and constraints upon the nurse is relieved. Further research into the barriers and opportunities to schools’ cooperation with SRH services in order to improve access to and utilization of SRH services by adolescents are also recommended.

## Additional file

Additional file 1: Focus Group Discussion guide (PDF 101 kb)

## Abbreviations

CHC: Community health centers
FGD: Focus group discussion
FP: Family planning
IUD: Intra-uterine device
MCH: Maternal and child healthcare
PAC: Post abortion care
PHC: Primary health clinics
SA: South Africa
SES: Socio-economic status
SRH: Sexual and reproductive healthcare
SSA: Sub-Saharan Africa
TOP: Termination of pregnancy
WHO: World Health Organization
