# Supplementary material for: Healthcare workers’ beliefs, motivations and behaviours affecting adequate provision of sexual and reproductive healthcare services to adolescents in Cape Town, South Africa: a qualitative study
Source: BMC Health Serv Res. 2018 Feb 13;18:109. doi: 10.1186/s12913-018-2917-0 (PMC5810035; doi:10.1186/s12913-018-2917-0)
Supplement: Supplementary file 1 — Focus Group Discussion guide (PDF 101 kb) [file 12913_2018_2917_MOESM1_ESM.pdf]

Additional File 1.

INTERVIEW GUIDE: FOCUS GROUP DISCUSSION

UNIVERSITY OF THE WESTERN CAPE

Private Bag X 17, Bellville 7535, South Africa

Tel: +27 21-959 2835/2283

1. Can you tell us about the SRH services provided for/to adolescents in this clinic?
2. How does your facility, in general deal with adolescents seeking sexual and reproductive healthcare services such as contraceptives for example, compared to adults?
    a. Do you think adolescents are dealt with differently from adults? Why? And why NOT?
    b. Do you deal with adolescents differently from adults? Why? And why NOT?
3. Overall, can you say you are please/happy/satisfied with the rate of adolescents coming to this clinic to seek SRH services?
    a. Why do you feel that way…?
    b. Do adolescents come here, to this facility for sexual and reproductive healthcare services?
    c. Why do you think they come here (those who come)?
    d. Why do you think they do NOT come here (those who do not come).

4. What do you think are the general opinions about adolescents' sexual activity in this facility? Probe for more information.
    a. Can you tell me about your own personally opinions about adolescents' sexual activity, in general.
5. Do you think one's personal opinions (feelings) about adolescents' sexual activity has an effect on the way they deal with adolescents seeking sexual and reproductive healthcare services, like family planning? Please elaborate
    a. Do you think your own personal opinions (feelings) about adolescents' sexual activity have an effect on how you deal with adolescents seeking sexual and reproductive healthcare services, like family planning? Please elaborate
6. Do you think there are certain situations/circumstances (motivations) where you would provide sexual and reproductive healthcare services to adolescents?
    a. Please explain
7. Are there certain situations/circumstances (motivations) where you would NOT provide sexual and reproductive healthcare services to adolescents?
    a. Please explain
8. Do you think certain things/circumstances in the facility (around you) facilitate these situations?
    a. Please elaborate
9. Are there any obstacles or reasons for you, personally that discourage (prevent) you from providing sexual and reproductive healthcare services to adolescents?

    a. For example, do you have any beliefs (religious reasons) that do not permit you to provide sexual and reproductive healthcare services to adolescents? Please explain
10. Are there any reasons for you, personally that encourage (promote) you to provide sexual and reproductive healthcare services to adolescents?
    a. For example, what makes you more willing (motivates you) to provide sexual and reproductive healthcare services to adolescents? Please explain
11. Do you think you, personally, have other reasons that might prevent you from providing sexual and reproductive healthcare services such as family planning to adolescents?
    a. Example, your colleagues and peers do not provide sexual and reproductive healthcare services to adolescents…?
12. Can you describe how you would response to a teenage asking you for condoms/ a morning after pill/abortion
13. Do you have any beliefs or traditions related to adolescents' sexual activity?
    a. Please explain
14. Is there anything you would like to add, suggest or recommend for the SRH services in this facility?
